# Freshwater Fish Siberian Dace Ingest Microplastics in the Remote Yenisei Tributary

**DOI:** 10.3390/toxics11010038

**Published:** 2022-12-30

**Authors:** Yulia Frank, Danil Vorobiev, Abhishek Mandal, Yana Lemeshko, Svetlana Rakhmatullina, Gopala Krishna Darbha

**Affiliations:** 1 Biological Institute, Tomsk State University, Lenina Ave. 36, Tomsk 634050, Russia; 2 Environmental Nanoscience Laboratory, Department of Earth Sciences, Indian Institute of Science Education and Research Kolkata, Mohanpur 741246, West Bengal, India

**Keywords:** microplastic ingestion, freshwater fish, gastrointestinal tract, the Yenisei basin

## Abstract

This study analyzed microplastics in the gastrointestinal tract of Siberian dace (*Leuciscus leuciscus* subsp. *baicalensis* (Dybowski, 1874)) in the remote Yenisei tributary of the Nizhnyaya (Lower) Tunguska River (Siberia, Russia). µRaman analysis showed that 60% of the fish from two different sites had ingested plastic microparticles (on average, 1.55 ± 1.95 items per individual). The most common type of microplastic were fibers, and the most abundant size category was 300 to 1000 µm. In the studied population, no significant differences in the MP content between the two sites or between males and females were found (*p* > 0.05). The tendency for higher MP ingestion by Siberian dace at earlier ages (2+ and 3+) compared to later (4+ and 5+) was observed, which may be connected to the features of the fish biology and ecology.

## 1. Introduction

Artificial plastics are widely used in all aspects of human life; many advances in medicine, technology, and industry would not have been possible without these materials. However, their relative availability, low cost, and difficulty in reuse have led to worldwide misuse with macro- and microplastic pollution [1]. Microplastics (MPs) are any plastic particles smaller than 5 mm along the maximum axis [2], which are either specially made to be micro-sized (primary MPs) or are formed as a result of the fragmentation of larger plastic items in the environment (secondary MPs) [3]. Artificial polymer microparticles are accumulating around the world at an increasing rate, especially in the aquatic environment [4,5].

Living organisms, including humans, are important receptors of MPs and can uptake tiny plastic particles via water, air, and food items [6]. MPs can be ingested by aquatic organisms from different trophic levels and transferred along aquatic food chains [7]. A significant amount of micro- and nanoplastics in water bodies are consumed by invertebrates and fish, resulting in their trophic transfer [8,9]. Natural biota in aquatic environments may ingest MPs, confusing them with food, many of which are linked to humans via food chains [10]. The bio-availability and the extent of toxicity are determined by their stability in the water column [11] and the associated contaminants in the vicinity [12]. As MPs entail potential risks to human health [6,7,13], it is necessary to investigate the mechanisms and scales of particle transfer in aquatic food chains. While the distribution of MPs in water, bottom sediments, and soils is being intensively studied, there is still no comprehensive monitoring of micro- and nanoplastic particles in food products. The volumes of MP consumption with sea fish and seafood are the most studied now. Analyzing global data, Jin et al. [14] found that aquatic food products present up to dozens plastic items per g for bivalves and per individual for fish. It was stated that up to 212 MP particles can enter the human body in this way every day [15]. Although the majority of ingested particles (>90%) are reported to be excreted from the body, particles smaller than 150 µm can translocate through the intestinal epithelium and enter the bloodstream, while particles smaller than 1.5 µm can penetrate tissues and organs [16].

Studies showed that freshwater fish, along with marine organisms, tend to ingest submerged MPs. According to Galafassi et al. [17], more than 250 species of freshwater fish have been documented to ingest MPs. Plastic microparticle ingestion has been detected in 32 countries, with presence in the digestive tract in more than 50% of the specimens analyzed in one study out of two. Judging by the world map of wildlife interactions with marine and freshwater litter [18], there are no data on the uptake and accumulation of MPs by fish in the rivers and lakes in Siberia. At the same time, fishing is a traditional activity of the local population and an important source of food as well [19].

The present study is focused on the quantitative assessment and characterization of MPs from the gastrointestinal tract (GIT) of freshwater fish, which serve as a food resource. Original experimental data were obtained for the Siberian dace (*Leuciscus leuciscus* subsp. *baicalensis* (Dybowski, 1874)) from the remote Yenisei tributary, the Nizhnyaya Tunguska, where fish is an important part of the local diet. This is also the first quantification of MP uptake by fish in the Yenisei basin; therefore, the obtained data will be useful for comparative assessments across different regions.

## 2. Materials and Methods

### 2.1. Study Object and Area

**Figure 1:**
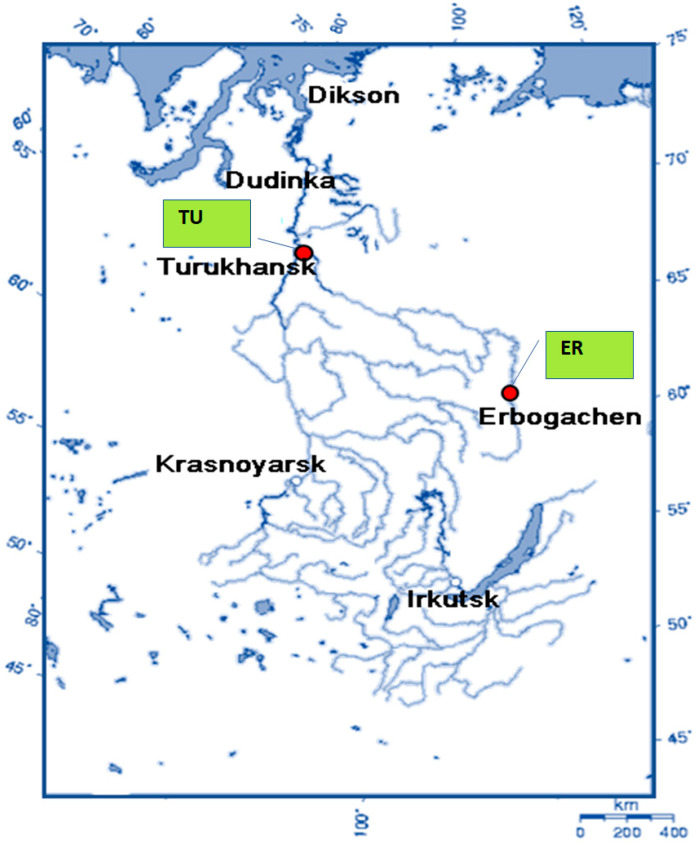
The Yenisei basin and sampling locations on the Nizhnyaya Tunguska (shown by red circles). Map source: Geo Swan, via Wikimedia Commons under a Creative Commons Attribution-Share Alike 3.0 Unported.

The object of the study was dace from the Nizhnyaya (N.) Tunguska or Lower Tunguska River, a large right tributary of the Yenisei in Siberia (Figure 1). In the territory of the Russian Federation, there are two subspecies of dace—*Leuciscus leuciscus* subsp. *leuciscus* (Linnaeus, 1758), and, East of the Ural Mountains, Siberian subspecies—*L. leuciscus* subsp. *baicalensis* (Dybowski, 1874) which is sometimes considered as *L. baicalensis* [20]. In this study, we studied *L. leuciscus* subsp. *baicalensis*, which is a widespread omnivorous species of the cyprinid family in the rivers and lakes of Siberia, including the lower Yenisei River [21].

Having a length of 3000 km, the N. Tunguska is the longest Yenisei tributary flowing from the Central Siberian Plateau. The catchment of the river is poorly populated. A more detailed description of the N. Tunguska River has been given previously [22]. Twenty individuals of *L. leuciscus* subsp. *baicalensis* were caught with a rod in late June to early July, 2021 in each of two sites: ‘ER’ near Erbogachen settlement in the Irkutsk region (61°17′00″ N 108°00′20″ E) and ‘TU’ near Turukhansk in the Krasnoyarsk region (65°47′13″ N 87°58′54″ E) (Figure 1).

### 2.2. Biological Analysis of the Fish and Sample Preparation

Each of the 40 fish individuals were measured to the nearest 0.5 mm to determine the total length (L) and the standard length (l) and weighed for the total weight (Q) and body weight without viscera (q), ±0.5 g. The fish age was determined by the number of annual rings on the fish scales taken in the region of the dorsal fin. The scales were cleaned using a 4% solution of ammonia, prepared for study by mounting the whole scales on glass slides and examining under a dissecting microscope. The age of a fish was determined by counting the number of annuli or year marks using an anterior diagonal scale radius [23].

The fish samples were ventrally dissected for further processing. The sex of the fish was determined visually by their gonads [24]. The gastrointestinal tract (GIT), including the esophagus, stomach, and intestine, was removed as described by [25] and fixed in 70% ethanol until laboratory analyses [26].

### 2.3. Microplastic Extraction, Detection, and Quantification

To extract MPs from the GITs, alkaline hydrolysis in KOH with the following density separation was carried out based on the modified protocol that was described for mussels by Jahan et al. [27]. Hydrolysis of each GIT was carried out individually in a glass beaker with 200 mL of 10% KOH, followed by stirring the sample on a shaker at a temperature of 55 °C for 48 h. After tissue destruction, MP particles were extracted by density separation in a saturated NaCl solution (~1.20 g cm^−3^) overnight. To eliminate the products of fat saponification, the upper phase from the separating funnel was additionally treated with 96% ethanol (10% of the sample volume) [28].

Then, the upper fraction was vacuum-filtered using 0.45 μm mixed cellulose ester membranes (MF-Millipore, Darmstadt, Germany). Preliminary visual inspection of the filters was conducted using light microscopy (stereomicroscope Micromed MC2, Saint Petersburg, Russia) with a digital camera and ToupView 3.7.6273 software. Particles were captured using a camera for the records. MPs from fish GITs were analyzed using a Raman spectrometer (LabRam 800; Horiba Jobin-Yvon, GmbH, Bensheim, Germany) in backscattering geometry with CCD as a detector. A green laser of 532 nm was used as an excitation source, and a grating with 1800 g/mm was used as a monochromator. The laser spot was focused on the target with a 50× objective. Where necessary, a baseline correction was applied. The obtained spectra were then compared with that of the known plastic materials publicly available in PublicSpectra.

The plastic items were categorized into groups by their shape as described by Frias and Nash [29]: films, fibers, and irregular shaped fragments. The particles were also classified by their major dimension into six groups: 150–300, 300–1000, 1000–2000, 2000–3000, 3000–4000, and 4000–5000 µm. Abundance of MPs above 150 µm was evaluated using arithmetical mean ± SD; percentages of different particle type occurrence in two sampled populations (ER and TU) were also calculated.

Both samples (ER and TU) corresponded to the conditions of a normal distribution as was determined using the skewness and kurtosis measures. The Student test [30] was used to compare differences in biological parameters and MP abundance in GITs. To compare differences in MPs counts between fish of different ages, a non-parametric Mann–Whitney U test [31] was used. In both cases, differences at *p* ≤ 0.05 were considered statistically significant. The correlation in biological characteristics and MPs counts was evaluated using Pearson’s correlation coefficient [32].

### 2.4. Contamination Prevention and Control Procedures

To prevent the samples’ contamination with MPs, the materials and implements for field and laboratory processing were made of stainless steel, glass, and aluminum where it was possible; cotton clothing was used in laboratory and the field. Airborne contamination in the sample preparation process was controlled using replicated procedural blanks of negative controls (*n* = 3 for each batch of samples) as recommended [33]. The filters with the MPs were stored in clean glass Petri dishes that were covered with lids after filtration and prepared for microscopic observation.

## 3. Results

### 3.1. Biological Characteristics of Siberian dace from the N. Tunguska River

Forty individuals of *L. leuciscus* subsp. *baicalensis* (Siberian dace) from the Yenisei tributary, the Nizhnyaya Tunguska River, were analyzed. The average linear–weight characteristics of dace caught in the two distant sites are presented in Table 1.

Among the examined individuals, 24 were females, and 16 were males. The age of the fish in the studied populations ranged from 3+ to 6+ years and was distributed as follows: 3+ group included 5 individuals, 4+ and 5+ groups consisted of 16 individuals each, 3 individuals were of 6+ age.

### 3.2. Microplastic Abundance in Gastrointestinal Tracts of the Fish

Preliminary microscopic analysis revealed a total of 71 microplastic-like particles. Of these, for 62 pieces (87.3%), the polymer composition was confirmed by µRaman. For all 40 analyzed fish individuals, the average MP content in GITs was 1.55 ± 1.95 items ind^−1^ or 43.1 ± 52.5 items kg^−1^; plastic was detected in 60.0% of Siberian dace. MPs were found in 15 out of 20 analyzed individuals from the ER site, which is 75.0% (Table 2). For dace caught at the mouth of the river, the TU site, this was 45.0% (9 out of 20 analyzed individuals contained MPs in the GIT). On average, 1.00 ± 1.38 MP items accounted for one dace (48.0 ± 56.4 items kg^−1^) at the mouth of the N. Tunguska River and 2.10 ± 2.29 items per individual (38.1 ± 49.3 items kg^−1^) detected upstream, in the ER site (Table 2). Analysis of the quantitative data using the Student’s *t*-test showed that there are no differences in the content of MPs in the fish GITs between dace from the ER and TU sites (*p* > 0.05).

**Figure 2:**
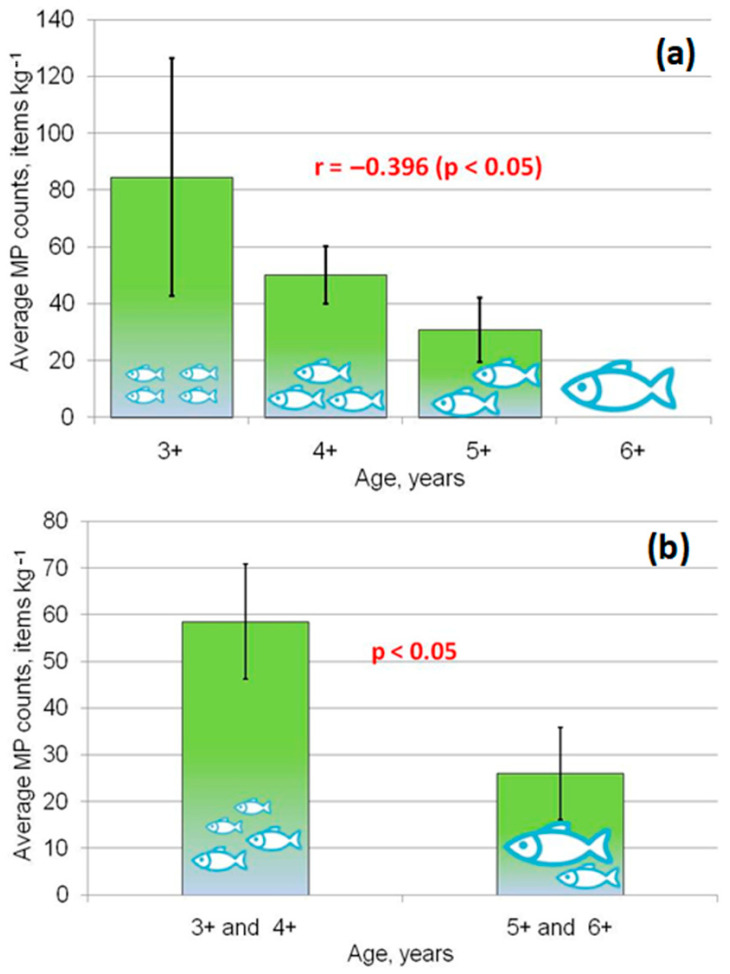
Ingestion of MPs in Siberian dace: (**a**) average MP counts in GITs of fish (arithmetic mean ± standard error) per 1 kg, r—Pearson’s correlation; (**b**) average MP counts in GITs of fish in younger and older age groups (arithmetic mean ± standard error) per 1 kg, Mann–Whitney U test.

The Student *t*-test revealed no significant differences in MP ingestion between males and females. The MP uptake by fish in the studied populations did not correlate with their linear–weight characteristics. A moderate negative Pearson’s correlation (r = −0.396, *p* < 0.05) between the average MP counts per kg and the age of dace individuals was noticed (Figure 2a). Differences in the MP ingestion between each of the ages (3+, 4+, 5+ and 6+) were not significant; however, when younger (3+ and 4+) and older (5+ and 6+) individuals were joined into two groups, significant differences in the average MP content per kg were revealed using the Mann–Whitney U test at *p* < 0.05 (Figure 2b).

### 3.3. Polymer Composition and Morphology of Microplastics

**Figure 3:**
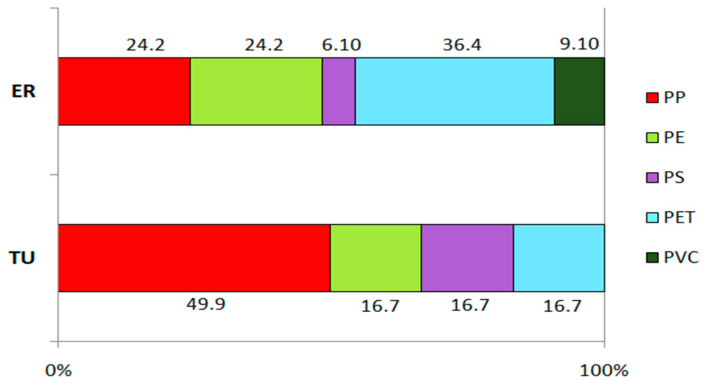
Polymer composition of the MPs detected in Siberian dace individuals collected in ER and TU sites.

Five types of plastic were recovered from Siberian dace GITs (Figure 3 and Figure S1). Polypropylene (PP) was the most common overall, accounting for 24.2 to 49.9% in fish from the ER and TU sites, correspondingly. Other particles were identified as polyethylene (PE) (24.2 and 16.7% for ER and TU samples), polystyrene (PS) (6.10 and 16.7%), and polyethylene terephthalate (PET) (36.4 and 16.7%). Polyvinyl chloride (PVC) was detected in the ER samples only and comprised up to 9.10% of all MPs (Figure 3).

**Figure 4:**
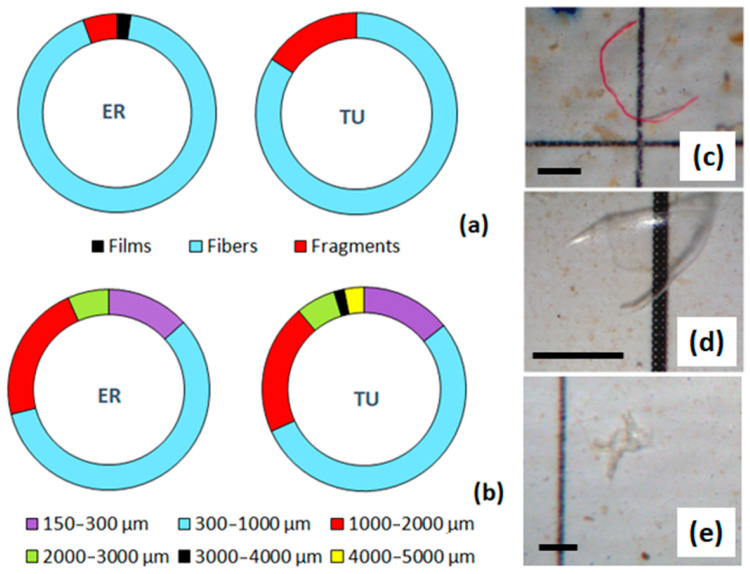
Morphology of MPs from the fish GITs: MP shape (**a**) and size (**b**) composition, %; microphotographs of the fiber (**c**), fragment (**d**), and film (**e**) extracted from Siberian dace individuals. Scale bar is 500 µm.

MPs extracted from Siberian dace individuals were classified into three categories by particle shape: fibers, films, and irregular shaped fragments (Figure 4). Elongated microfibers were the predominant type of MPs in both the ER and TU populations and made up to 92.2 and 84.1%, correspondingly (Figure 4a). The remaining particles were represented by fragments in the TU samples (15.9% of the total particle counts), as well as fragments (5.56%) and films (2.24%) in the ER samples.

MP items recovered from fish GITs ranged in length, whereas the most common size class consumed by Siberian dace was 300–1000 µm which made up more than a half of all particles, 57.8% for ER and 54.0% for TU (Figure 4b). The second largest category was 1000–2000 µm (22.2 and 20.6% for ER and TU, correspondingly), followed by particles of 150–300 µm (13.3 and 14.3%) and 2000–3000 µm (6.70 and 6.35%). Larger MPs (3000–4000 and 4000–5000 µm) were found in TU samples only and their proportion was as low as ~1–3% (Figure 4b).

## 4. Discussion

This study appears to be the first to describe the ingestion of MPs by freshwater fish in the Yenisei River basin. Plastic microparticles were confirmed in ~60% of Siberian dace individuals from the Yenisei remote tributary, Nizhnyaya Tunguska. Previously the presence of MPs in the surface waters and bottom sediments of the N. Tunguska and in the adjacent section of the Yenisei River was confirmed [22]. The concentration of MPs in the bottom sediments of the N. Tunguska reached 543 ± 94.1 items kg^−1^ in the zone of increased sedimentation; the average MP content in the water varied from 1.20 ± 0.70 near the Erbogachen settlement (ER) to 4.53 ± 2.04 items m^−3^ near Turukhansk (TU) and tended to rise in the watercourse. In the current study, we did not observe differences in the ingestion of MPs by fish in these two distant sites. The probable sources of MPs in the remote continental water bodies may include mismanaging plastic waste in settled areas, using synthetic fabrics, and the fishing activity of the tourists and local population [34,35,36,37]. The potential sources of MPs ingested by Siberian dace in the current study are the same as plastic pollution in the N. Tunguska and Yenisei Rivers is mainly associated with human activities, including plastic wasting, the use of synthetic fabrics, and intensive fishing [22].

Quantitative assessments of MP ingestion by freshwater fish were also carried out for other regions in the world [17,38,39]. MPs were found in the GIT of <50% of all studied fish specimens from the River Thames, Great Britain [40], urban wetlands in Australia [41], and the Amazon estuary [42]. MPs were found in half of the fish GITs from the Pearl River (*n* = 279) [43]. More than 90% of fish from catches in the tributaries of the River Michigan, USA, and from Lake Taihu, China, ingested synthetic microparticles [44,45]. In small samples (up to 20 individuals) from the Bahía Blanca estuary, Argentina, and the Tom River, Western Siberia, Russia, MPs were detected in the GIT of 100% examined fish [46,47]. Published data on the average content of MPs in the GITs of different species of freshwater fish inhabiting Asian rivers vary widely from <1 to several tens of particles per individual [38,39]. The highest concentrations of MPs were recorded in the fish of the rivers of Southeast Asia with the maximum values of 22 ± 16 and 14–94 items ind^−1^ in the Khan River, South Korea [48], and in the Fengshan River, northern Taiwan [49], correspondingly. The content of MPs in the gastrointestinal tract of fish from the N. Tunguska River detected in the current study (1.55 ± 1.95 items ind^−1^) is not high in comparison with the literature data. This is due both to the different levels of plastic pollution in rivers and the differences in the methodology used.

It is known that the ingestion and accumulation of MPs in the GIT of fish, in many cases, is associated with the lifestyle, nutrition, and trophic status of certain species. As bottom sediments are MPs concentrators, demersal fish are more prone to particle uptake compared to pelagic ones [38]. Several studies have confirmed a higher MP content in the GIT of demersal fish [44,50]. Undoubtedly, the position in the food chain and the type of feeding are associated with the absorption of MPs. However, the literature data are somewhat contradictory. Some researchers believe that predators or obligate piscivores are more prone to the ingestion and accumulation of MPs compared to species adhering to other feeding strategies due to particle magnification in food chains [38]. Other authors note that fish-eating predators, in contrast, accumulate fewer particles, as they consume them randomly or indirectly, together with the prey, whereas other species indiscriminately consume MPs along with the food [51]. Among the studied fish species of the Thames River, the maximum number of particles per individual was found in zoobenthophages *Neogobius melanostomus* in comparison with omnivorous fish and detritivores [50]. In other studies, the percentage of MP particles was higher for omnivorous fish [43,52]. Our data suggest plastic particle ingestion by Siberian dace was significantly higher at earlier ages (2+ and 3+) compared to later (4+ and 5+). This may be connected to the change in types of diet with age when they begin to prey more often or related to other ecological and biological features of the fish and requires further research.

The characteristics of MPs extracted from the dace GITs in the current study were similar to those determined earlier for the water and bottom sediments. Microfibers, microfragments of irregular shape, and microfilms (in descending order) were detected among the plastic particles from the N. Tunguska [22]. Fibers were the dominating MPs in the GITs of Siberian dace from the TU and ER sites along the N. Tunguska River and made up >80 and >90% of the total counts, correspondingly. Plastic microfragments and microfilms but no spheres were detected in fish GITs as well as in the water and sediments of the river. Meta-analysis recently showed that the largest percentage of MPs ingested by fish was in the form of fibers and fragments [53].

Fibers are plastics that have been spun into fibers or filaments and used to make fabrics, string, ropes, and cables [54]. One of the most recognizable plastic fibers is polyester (polyethylene terephthalate, PET), which was identified among MPs from Siberian dace GITs. The predominant accumulation of synthetic fibers in the GIT of fish, identified in the current study and by a number of authors, can be explained by the fact that freshwater ecosystems are largely prone to pollution by wastewater and effluents from water treatment facilities. Fibers in the environment originate mainly from the effluent of wastewater treatment plants [55,56]. In addition, fishing in many regions is the cause of secondary fiber pollution due to the degradation of lost and discarded fishing gear [3]. In the model experiments of Qiao et al. [57], when comparing the bioaccumulation of different forms of MPs in *Danio rerio*, the predominant accumulation of fibers was shown in the following order: fibers > fragments > granules. The more intensive accumulation of fibers was detected in *Carassius auratus* after the simultaneous introduction of MP fibers, spheres, and fragments into the diet [58]. This may be due to the fact that non-spherical forms of MPs (fibers, fragments) stay in the gastrointestinal tract of fish for a longer time than microspheres. In addition, due to the shape, fibers bind to tissues more easily than particles of other shapes, which subsequently leads to a more intense accumulation of MPs in the gastrointestinal tract and tissues [57,58].

The results of the current study confirm the ingestion of MPs by Siberian dace in remote regions such as the Yenisei tributary, the Nizhnyaya Tunguska River. Most of the recent studies on the assessment of MP uptake and accumulation in fish are focused on the detection of particles in the GIT. However, due to the fact that MPs can also get transferred in various organs through the bloodstream and lymph [16], they have been detected in the gills, liver, muscle tissue, gonads, and brain of freshwater fish [59,60,61]. These findings confirm the global significance of plastic pollution in the aquatic environment and highlights the need for quantitative assessments of MP ingestion, accumulation, and trophic transfer in freshwater food resources.

## Figures and Tables

**Table 1:** Biological characteristics of Siberian dace from the Nizhnyaya Tunguska River.

Site	N	L (mm)	l (mm)	Q (g)	q (g)	m(GIT) (g)
ER	20	179.9 ± 17.6	158.9 ± 12.4	48.33 ± 11.50	42.75 ± 10.10	2.99 ± 1.25
TU	20	152.2 ± 15.1	136.8 ± 12.9	30.21 ± 9.51	26.34 ± 8.25	1.73 ± 0.65

Note: data are presented as arithmetic mean ± standard deviation.

**Table 2:** MP content in the GITs of Siberian dace from the Nizhnyaya Tunguska River.

Site	N	% of Individuals with MPs	Av. ^1^ MP Content, Items ind^−1^	Av.^1^ MP Content, Items kg^−1^
ER	20	75.0	2.10 ± 2.29	48.0 ± 56.4
TU	20	45.0	1.00 ± 1.38	38.1 ± 49.3
Both sites	40	60.0	1.55 ± 1.95	43.1 ± 52.5

^1^ Arithmetic mean ± standard deviation.

## Data Availability

Not applicable.

## Supplementary Materials

The following supporting information can be downloaded at: https://www.mdpi.com/article/10.3390/toxics11010038/s1, Figure S1: Raman spectra of the polymers.

## References

[1] Horton A.A. (2022). Plastic pollution: When do we know enough?. J. Hazard. Mater..

[2] Arthur C., Baker J., Bamford H. (2009). Proceedings of the International Research Workshop on the Occurrence, Effects, and Fate of Microplastic Marine Debris, Tacoma, WA, USA, 9–11 September 2008.

[3] Boucher J., Friot D. (2017). Primary Microplastics in the Oceans: A Global Evaluation of Sources.

[4] Koutnik V.S., Leonard J., Alkidim S., DePrima F.J., Ravi S., Hoek E.M., Mohanty S.K. (2021). Distribution of microplastics in soil and freshwater environments: Global analysis and framework for transport modeling. Environ. Pollut..

[5] Bank M.S., Hansson S.V., Bank M.S. (2022). The microplastic cycle: An introduction to a complex issue. Microplastic in the Environment: Pattern and Process, Environmental Contamination Remediation and Management.

[6] Prata J.C., da Costa J.P., Lopes I., Duarte A.C., Rocha-Santos T. (2020). Environmental exposure to microplastics: An overview on possible human health effects. Sci. Total Environ..

[7] Huang W., Song B., Liang J., Niu Q., Zeng G., Shen M., Deng J., Luo Y., Wen X., Zhang Y. (2021). Microplastics and associated contaminants in the aquatic environment: A review on their ecotoxicological effects, trophic transfer, and potential impacts to human health. J. Hazard. Mater..

[8] Chae Y., Kim D., Kim S.W., An Y. (2018). Trophic transfer and individual impact of nano-sized polystyrene in a four-species freshwater food chain. Sci. Rep..

[9] Lehel J., Murphy S. (2021). Microplastics in the food chain: Food safety and environmental aspects. Rev. Environ. Contam. Toxicol..

[10] Cássio F., Batista D., Pradhan A. (2022). Plastic interactions with pollutants and consequences to aquatic ecosystems: What we know and what we do not know. Biomolecules.

[11] Abdolahpur Monikh F., Chupani L., Smerkova K., Bosker T., Cizar P., Krzyzanek V., Richtera L., Franek R., Zuskova E., Skoupy R. (2020). Engineered nanoselenium supplemented fish diet: Toxicity comparison with ionic selenium and stability against particle dissolution, aggregation and release. Environ. Sci. Nano.

[12] Singh N., Bhagat J., Tiwari E., Khandelwal N., Darbha G.K., Shyama S.K. (2021). Metal oxide nanoparticles and polycyclic aromatic hydrocarbons alter nanoplastic’s stability and toxicity to zebrafish. J. Hazard. Mater..

[13] Yuan Z., Nag R., Cummins E. (2022). Human health concerns regarding microplastics in the aquatic environment—From marine to food systems. Sci. Total Environ..

[14] Jin M., Wang X., Ren T., Wang J., Shan J. (2021). Microplastics contamination in food and beverages: Direct exposure to humans. J. Food Sci..

[15] Rubio-Armendáriz C., Alejandro-Vega S., Paz-Montelongo S., Gutiérrez-Fernández Á.J., Carrascosa-Iruzubieta C.J., Hardisson-de la Torre A. (2022). Microplastics as Emerging Food Contaminants: A Challenge for Food Safety. Int. J. Environ. Res. Public Health.

[16] EFSA (2016). Presence of microplastics and nanoplastics in food, with particular focus on seafood. EFSA J..

[17] Galafassi S., Campanale C., Massarelli C., Uricchio V.F., Volta P. (2021). Do freshwater fish eat microplastics? A review with a focus on effects on fish health and predictive traits of MPs ingestion. Water.

[18] Tekman M.B., Gutow L., Macario A., Haas A., Walter A., Bergmann M. Alfred Wegener Institute Helmholtz Centre for Polar and Marine Research. Literbase. https://litterbase.awi.de/litter.

[19] Sirina A.A. (2018). The folks next door. Russian settlers and Evenki of the upper flow of the Lower Tunguska (19th-early 21st century). Études Mong. Sib. Cent. Tibét..

[20] Kottelat M., Freyhof J. (2007). Handbook of European Freshwater Fishes.

[21] Lobón-Cerviá J., Dgebuadze Y., Utrilla C.G., Rincón P.A., Granado-Lorencio C. (1996). The reproductive tactics of dace in central Siberia: Evidence for temperature regulation of the spatio-temporal variability of its life history. J. Fish Biol..

[22] Frank Y.A., Vorobiev D.S., Kayler O.A., Vorobiev E.D., Kulinicheva K.S., Trifonov A.A., Soliman Hunter T. (2021). Evidence for microplastics contamination of the remote tributary of the Yenisei River, Siberia—The pilot study results. Water.

[23] Hile R. (1936). Age determination of fish from scales; Method and application to fish cultural problems. Progress. Fish-Cult..

[24] Pravdin I.F. (1966). Guide for the Study of Fish.

[25] Khan F.R., Shashoua Y., Crawford A., Drury A., Sheppard K., Stewart K., Sculthorp T. (2020). ‘The Plastic Nile’: First Evidence of Microplastic Contamination in Fish from the Nile River (Cairo, Egypt). Toxics.

[26] Capone A., Petrillo M., Misic C. (2020). Ingestion and elimination of anthropogenic fibres and microplastic fragments by the European anchovy (*Engraulis encrasicolus*) of the NW Mediterranean Sea. Mar. Biol..

[27] Jahan S., Strezov V., Weldekidan H., Kumar R., Kan T., Sarkodie A.S., He J., Dastjerdi B., Wilson S.P. (2019). Interrelationship of microplastic pollution in sediments and oysters in a seaport environment of the eastern coast of Australia. Sci. Total Environ..

[28] Dawson A.L., Motti C.A., Kroon F.J. (2020). Solving a sticky situation: Microplastic analysis of lipid-rich tissue. Front. Environ. Sci..

[29] Frias J.P.G.L., Nash R. (2019). Microplastics: Finding a consensus on the definition. Mar. Pollut. Bull..

[30] Student (1908). The probable error of a mean. Biometrika.

[31] Mann H.B., Whitney D.R. (1947). On a test of whether one of two random variables is stochastically larger than the other. Ann. Math. Stat..

[32] Kirch W., Kirch W. (2008). Pearson’s Correlation Coefficient. Encyclopedia of Public Health.

[33] Koelmans A.A., Nor N.H.M., Hermsen E., Kooi M., Mintenig S.M., De France J. (2019). Microplastics in freshwaters and drinking water: Critical review and assessment of data quality. Water Res..

[34] Frank Y.A., Vorobiev E.D., Vorobiev D.S., Trifonov A.A., Antsiferov D.V., Soliman Hunter T., Wilson S.P., Strezov V. (2021). Preliminary screening for microplastic concentrations in the surface water of the Ob and Tom Rivers in Siberia, Russia. Sustainability.

[35] van der Wal M., van der Meulen M., Tweehuijsen G., Peterlin M., Palatinus A., Kovač Viršek M., Coscia L., Kržan A. (2015). Identification and Assessment of Riverine Input of (Marine) Litter.

[36] Free C.M., Jensen O.P., Mason S.A., Eriksen M., Williamson N., Boldgiv B. (2014). High-levels of microplastic pollution in a large, remote, mountain lake. Mar. Pollut. Bull..

[37] Jiang C., Yin L., Li Z., Wen X., Luo X., Hu S., Yang H., Long Y., Deng B., Huang L. (2019). Microplastic pollution in the rivers of the Tibet Plateau. Environ. Pollut..

[38] Collard F., Gasperi J., Gabrielsen G.W., Tassin B. (2019). Plastic particle ingestion by wild freshwater fish: A critical review. Environ. Sci. Technol..

[39] Parvin F., Jannat S., Tareq S.M. (2021). Abundance, characteristics and variation of microplastics in different freshwater fish species from Bangladesh. Sci. Total Environ..

[40] Horton A.A., Jürgens M.D., Lahive E., van Bodegom P.M., Vijver M.G. (2018). The influence of exposure and physiology on microplastic ingestion by the freshwater fish *Rutilus rutilus* (roach) in the River Thames, UK. Environ. Pollut..

[41] Su L., Nan B., Hassell K.L., Craig N.J., Pettigrove V. (2019). Microplastics biomonitoring in Australian urban wetlands using a common noxious fish (*Gambusia holbrooki*). Chemosphere.

[42] Pegado T.S.E.S., Schmid K., Winemiller K.O., Chelazzi D., Cincinelli A., Dei L., Giarrizzo T. (2018). First evidence of microplastic ingestion by fishes from the Amazon River estuary. Mar. Pollut. Bull..

[43] Zheng K., Fan Y., Zhu Z., Chen G., Tang C., Penga X. (2019). Occurrence and species-specific distribution of plastic debris in wild freshwater fish from the Pearl River catchment, China. Environ. Toxicol. Chem..

[44] Jabeen K., Su L., Li J.N., Yang D.Q., Tong C.F., Mu J.L., Shi H.H. (2017). Microplastics and mesoplastics in fish from coastal and fresh waters of China. Environ. Pollut..

[45] McNeish R.E., Kim L.H., Barrett H.A., Mason S.A., Kelly J.J., Hoellein T.J. (2018). Microplastic in riverine fish is connected to species traits. Sci. Rep..

[46] Arias A.H., Ronda A.C., Oliva A.L., Marcovecchio J.E. (2019). Evidence of microplastic ingestion by fish from the Bahía Blanca Estuary in Argentina, South America. Bull. Environ. Contam. Toxicol..

[47] Frank Y.A., Vorobiev E.D., Babkina I.B., Antsiferov D.V., Vorobiev D.S. (2020). Microplastics in fish gut, first records from the Tom River in West Siberia, Russia. Vestn. Tomsk. Gos. Univ. Biol..

[48] Park T.-J., Lee S.-H., Lee M.-S., Lee J.-K., Lee S.-H., Zoh K.-D. (2020). Occurrence of microplastics in the Han River and riverine fish in South Korea. Sci. Total Environ..

[49] Tien C.J., Wang Z.X., Chen C.S. (2020). Microplastics in water, sediment and fish from the Fengshan River system: Relationship to aquatic factors and accumulation of polycyclic aromatic hydrocarbons by fish. Environ. Pollut..

[50] McGoran A.R., Clark P.F., Morritt D. (2017). Presence of microplastic in the digestive tracts of European flounder, *Platichthys flesus*, and European smelt, *Osmerus eperlanus*, from the River Thames. Environ. Pollut..

[51] Wesch C., Bredimus K., Paulus M., Klein R. (2016). Towards the suitable monitoring of ingestion of microplastics by marine biota: A review. Environ. Pollut..

[52] Wang S., Zhang C., Pan Z., Sun D., Zhou A., Xie S., Wang J., Zou J. (2020). Microplastics in wild freshwater fish of different feeding habits from Beijiang and Pearl River Delta regions, south China. Chemosphere.

[53] Lim K.P., Lim P.E., Yusoff S., Sun C., Ding J., Loh K.H. (2022). A Meta-Analysis of the characterisations of plastic ingested by fish globally. Toxics.

[54] Elias S., Elias S.A. (2021). Impacts of chemical pollution on marine ecosystems. Threats to the Arctic.

[55] Murphy F., Ewins C., Carbonnier F., Quinn B. (2016). Wastewater treatment works (WwTW) as a source of microplastics in the aquatic environment. Environ. Sci. Technol..

[56] Ziajahromi S., Neale P.A., Leusch F.D.L. (2016). Wastewater treatment plant effluent as a source of microplastics: Review of the fate, chemical interactions and potential risks to aquatic organisms. Water Sci. Technol..

[57] Qiao R., Deng Y., Zhang S., Wolosker M.B., Zhu Q., Ren H., Zhang Y. (2019). Accumulation of different shapes of microplastics initiates intestinal injury and gut microbiota dysbiosis in the gut of zebrafish. Chemosphere.

[58] Jabeen K., Li B., Chen Q., Su L., Wu C., Hollert H., Shi H. (2018). Effects of virgin microplastics on goldfish (*Carassius auratus*). Chemosphere.

[59] Lu Y., Zhang Y., Deng Y., Jiang W., Zhao Y., Geng J., Ding L., Ren H. (2016). Uptake and accumulation of polystyrene microplastics in Zebrafish (Danio rerio) and toxic effects in liver. Environ. Sci. Technol..

[60] Ding J., Zhang S., Razanajatovo R.M., Zou H., Zhu W. (2018). Accumulation, tissue distribution, and biochemical effects of polystyrene microplastics in the freshwater fish red tilapia (*Oreochromis niloticus*). Environ. Pollut..

[61] Aryani D., Khalifa M.A., Herjayanto M., Solahudin E.A., Rizki E.M., Halwatiyah W., Istiqomah H., Maharani S.H., Wahyudin H., Pratama G. (2021). Penetration of microplastics (polyethylene) to several organs of Nile Tilapia (*Oreochromis niloticus*). IOP Conf. Ser. Earth Environ. Sci..

