# Supplementary material for: Freshwater Fish Siberian Dace Ingest Microplastics in the Remote Yenisei Tributary
Source: Toxics. 2022 Dec 30;11(1):38. doi: 10.3390/toxics11010038 (PMC9865856; doi:10.3390/toxics11010038)
Supplement: Supplementary file 1 [file toxics-11-00038-s001.zip › toxics-2071226-supplementary.pdf]

Yulia Frank, Danil Vorobiev, Abhishek Mandal, Yana Lemeshko, Svetlana Rakhmatullina and Gopala Krishna Darbha

Freshwater fish Siberian dace ingest microplastics in the remote Yenisei tributary

Supplementary Material

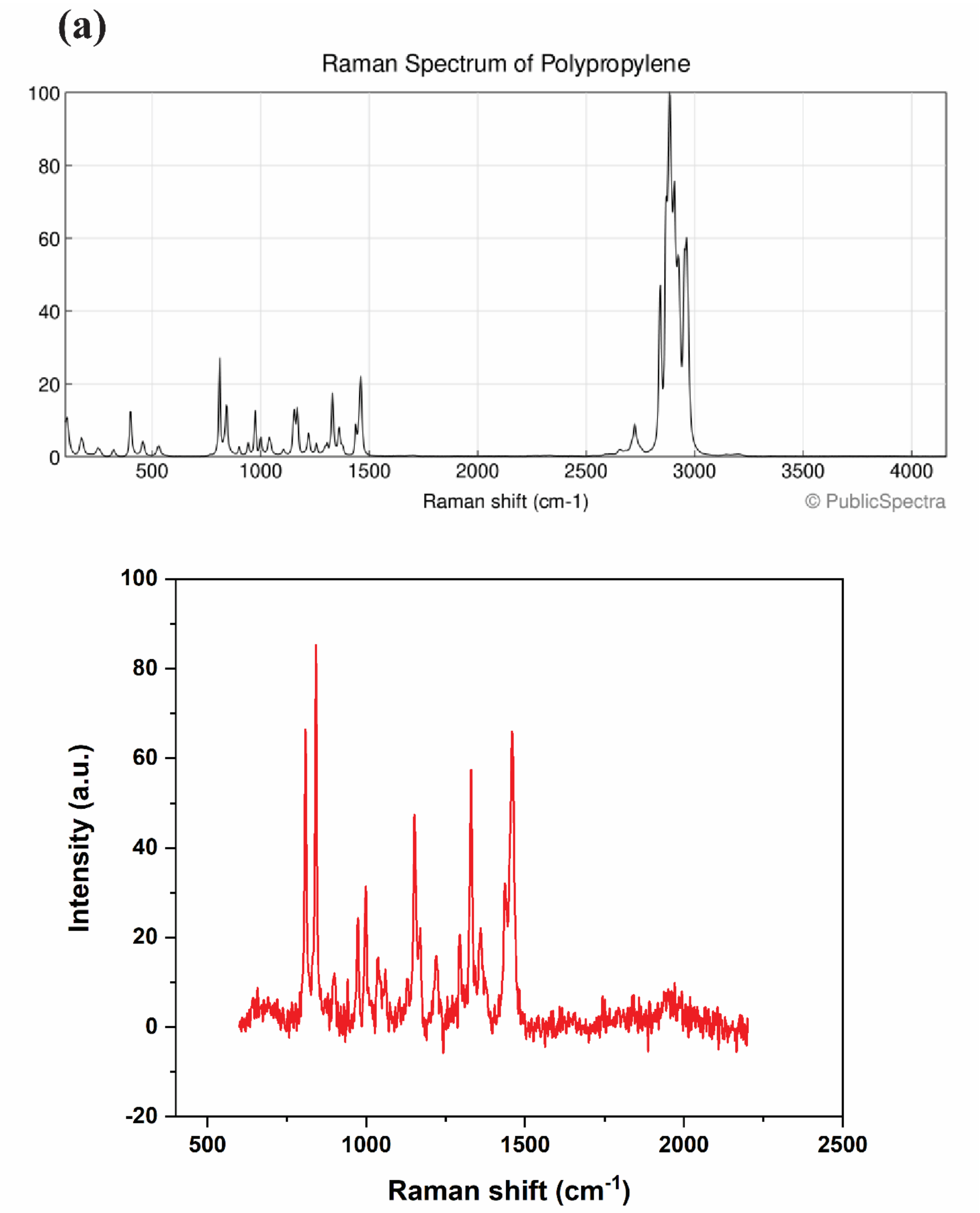

Figure S1. Raman spectra of polypropylene (Top shows the standard data from *PublicSpectra* and bottom shows spectra for plastic found in fish)

(b)

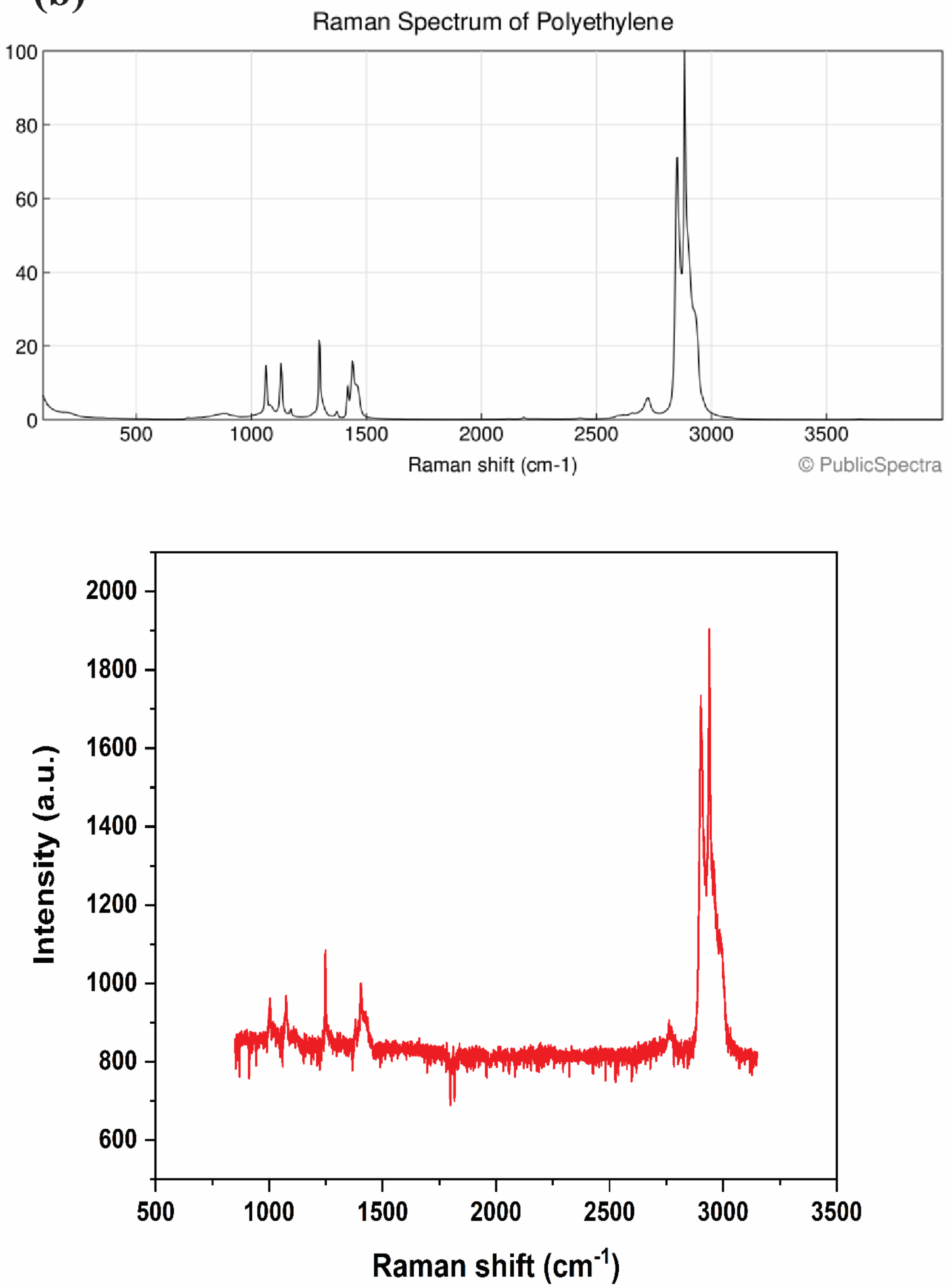

Figure S1 (continued). Raman spectra of polyethylene (Top shows the standard data from *PublicSpectra* and bottom shows spectra for plastic found in fish)

(c)

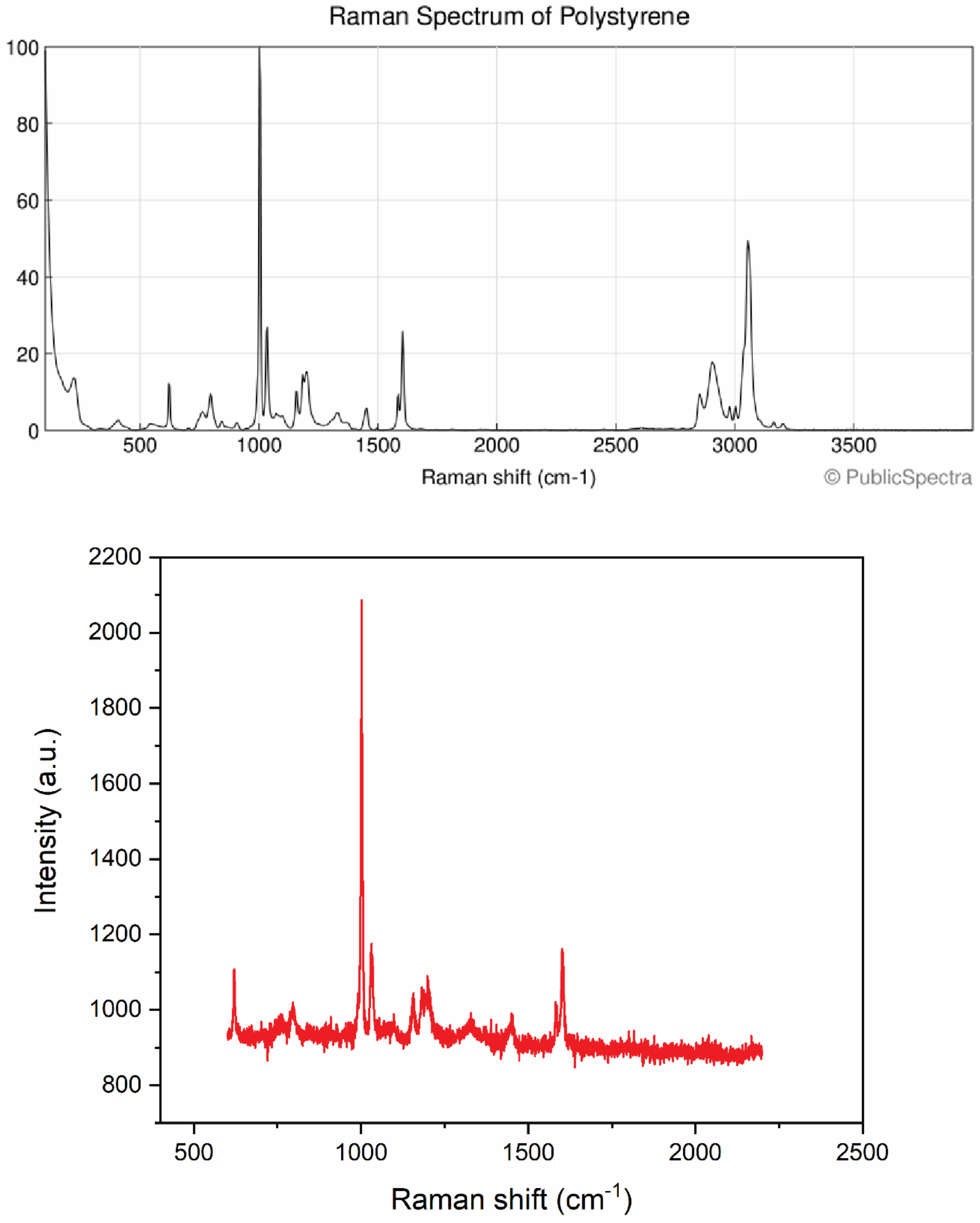

Figure S1 (continued). Raman spectra of polystyerene (Top shows the standard data from *PublicSpectra* and bottom shows spectra for plastic found in fish)

(d)

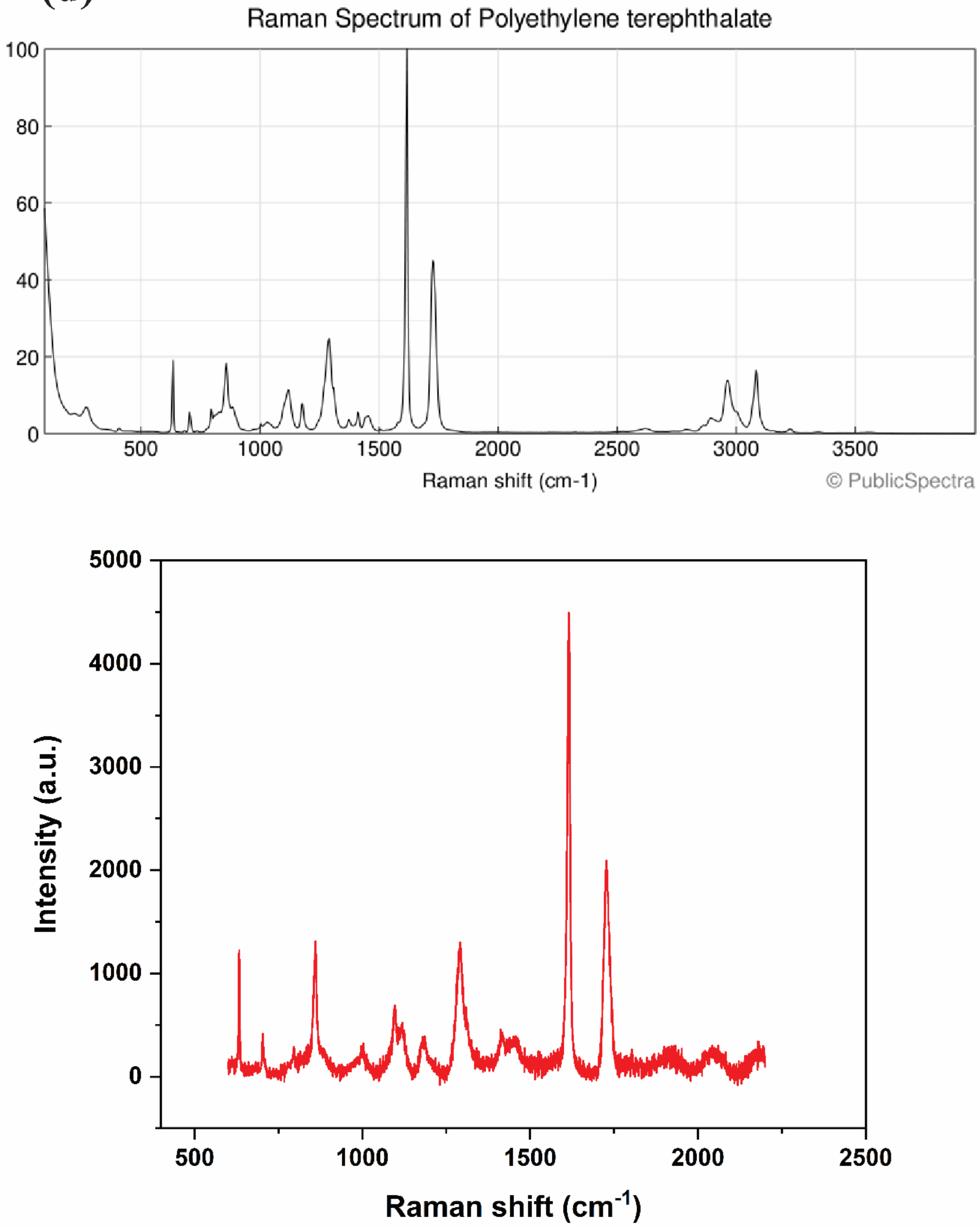

Figure S1 (continued). Raman spectra of polyethylene (Top shows the standard data from *PublicSpectra* and bottom shows spectra for plastic found in fish)

(e)

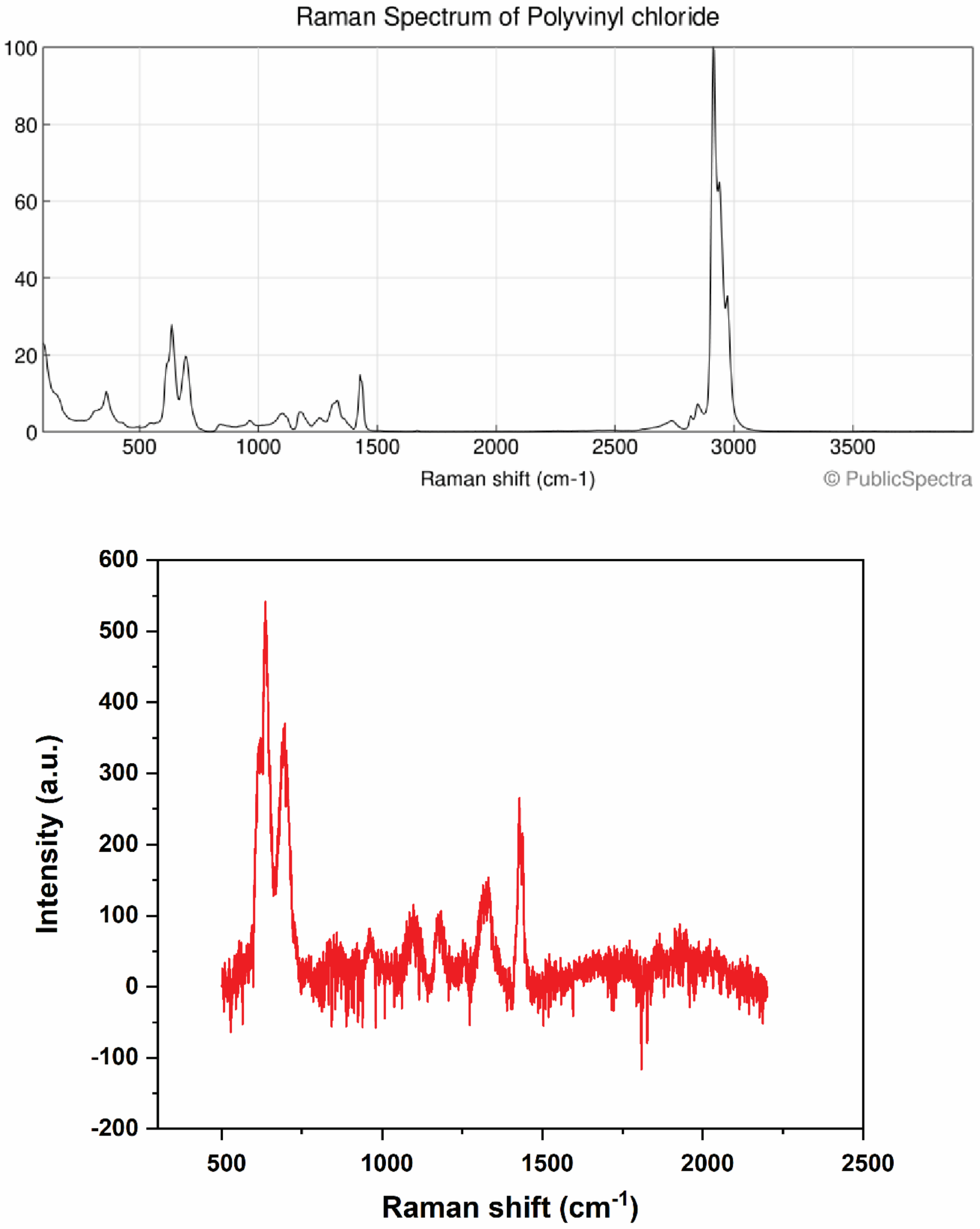

Figure S1 (end). Raman spectra of polyvinyl chloride (Top shows the standard data from *PublicSpectra* and bottom shows spectra for plastic found in fish)
